# CD8^+^ tissue-resident memory cells- do they modulate peripheral nervous system and neuroinflammation?

**DOI:** 10.3389/fimmu.2026.1933495

**Published:** 2026-09-14

**Authors:** Pushpa Pandiyan

**Affiliations:** 1 Department of Biological Sciences, School of Dental Medicine, Case Western Reserve University, Cleveland, OH, United States; 2 Department of Pathology, Department of Molecular Biology and Microbiology, School of Medicine, Center for AIDS Research, Case Western Reserve University, Cleveland, OH, United States

**Keywords:** mucosal, neuroimmune, neuroinflammation, peripheral nerve, TRM

## Abstract

CD8^+^ TRM cells are indispensable for continuous surveillance of tissues and direct elimination of invading pathogens through a diverse range of effector mechanisms. They balance adhesion, metabolic adaptation, and commensal tolerance against the capacity for swift, targeted attack, using local tissue signals and immunological memory as a finely calibrated control mechanism that prevents both chronic inflammation and persistent infection. Given their strategic co-localization with the peripheral nervous system (PNS) across multiple tissues, this raises a compelling question: do CD8^+^ TRM cells actively regulate peripheral nerve function? In light of recent evidence on how chronic HIV infection influences mucosal CD8^+^ TRM cells and tissue inflammation, this article critically evaluates the existing literature to assess the evidence and potential mechanisms underlying the involvement of CD8^+^ TRM cells in peripheral nerve inflammation across diverse tissue and disease contexts.

## Introduction

CD8^+^ tissue-resident memory T cells (TRM) cells represent a specialized memory T cell subset that establishes long-term, self-sustaining residence within specific tissues-distinct from recirculating central memory (TCM) and effector memory (TEM) counterparts that continuously traffic between blood, lymphoid organs, and peripheral tissues (1). CD8^+^TRM cells, hereafter referred to as TRM cells, permanently lodge themselves in barrier non-lymphoid tissues (NLTs), such as the oral mucosa, intestine, skin, lungs, and female reproductive tract-never to return to the bloodstream, which seems like a costly commitment of resources to a single location (2, 3). Yet this apparent inflexibility creates extraordinary responsiveness: by being pre-positioned exactly where pathogens enter, TRM cells can mount defenses within hours rather than days, acting as an early warning system that alerts other immune cells before threats spread systemically. The paradox deepens when one considers that these “stuck” cells maintain remarkable plasticity in their functional programs toggling between quiescence and explosive cytokine production and between tissue maintenance and tissue destruction (4, 5), dictated by local signals (6, 7). This combination of fixed positioning and dynamic responsiveness makes TRM cells uniquely suited not only for frontline defense against infection but also as potential mediators of immune-nerve interactions within peripheral nervous system-containing tissues.

## Functional markers and effector mechanisms of TRM cells

TRM cells are defined by core surface markers, primarily CD69 and CD103, which mediate tissue retention through distinct mechanisms (5). CD69 promotes residency by interacting with and sequestering the sphingosine-1-phosphate receptor 1 (S1PR1), antagonizing S1PR1 signaling, thereby preventing tissue egress. It also suppresses other migratory molecules including CD62L, CX3CR1, and KLF2. While CD69 can be transiently expressed on effector T cells shortly after activation in lymph nodes, its expression on TRM cells is typically constitutive and is not associated with other markers of recent T cell activation. Studies in mice have demonstrated that the genetic deletion of CD69 leads to a reduction, although not a complete elimination, of TRM cells in tissues such as the skin and lung. Importantly, in humans, the isolation of tissue memory T cells based on the expression of CD69 reveals a distinct transcriptional profile characteristic of TRM cells, further solidifying its role as a key identifier. CD103 (integrin αE) anchors TRM cells to epithelial surfaces via E-cadherin interactions, with its expression upregulated by TGF-β and predominantly observed in mucosal and barrier tissues. TRM cells residing outside of epithelial tissues generally do not express CD103 and this molecule has been shown to be important for survival of T cells in these compartments (8–12). Beyond these core markers, tissue-specific markers such as CD49a (VLA-1) and CXCR6 further define TRM subsets in skin and liver (13–15). While CD49a facilitates retention in mucosal tissues through collagen IV binding, CXCR6, a chemokine receptor binds to the chemokine CXCL16. The interaction between CXCR6 and CXCL16 is thought to promote the establishment and maintenance of TRM cells in these tissues (15). Programmed cell death-1 receptor (PD-1) is chronically expressed on brain TRM cells and a subset of TRM cells in other tissues like oral mucosa (6, 16–18). While PD-1 is often associated with T cell exhaustion in the context of chronic infections and tumors, its expression on brain TRM cells may serve as a mechanism to prevent excessive inflammation (6, 16–19). The expression of PD-1 on brain TRM cells has been shown to correlate with local antigen encounter. Inhibitory receptors including CTLA-4, TIM-3, and CD244 are also commonly expressed, reflecting a tightly regulated activation state shaped by the local tissue microenvironment.

Upon activation, TRM cells rapidly produce pro-inflammatory cytokines, including IFN-γ, TNF-α, and IL-2, initiating localized immune surveillance (20, 21). Tissue-specific cytokine profiles also emerge, such as IL-17 production by skin-resident TRM cells, including CD4^+^TRM cells (22, 23). Alongside cytokine production, TRM cells deploy potent cytotoxic machinery, releasing granzyme B and perforin to directly eliminate infected or transformed cells (13). Notably, CD103 engagement enhances cytolytic synapse formation, while CD49a-expressing skin TRM cells are primed for robust cytotoxic output upon IL-15 stimulation (13, 24). The co-expression of activation markers alongside inhibitory receptors like PD-1 and LAG-3 highlights the carefully balanced activation state of TRM cells, preventing immunopathology while maintaining rapid effector readiness (25, 26).

## Key transcription factors regulating TRM cell identity and function

The identity and tissue residency of TRM cells are governed by a core transcriptional network centered on Hobit and Blimp-1, which cooperate to suppress tissue egress genes such as CCR7 and S1PR1 while maintaining cytotoxic potential through granzyme B regulation, though their relative contributions vary by tissue and species (27). Runx3 serves as another critical regulator, establishing chromatin accessibility in non-lymphoid tissues and tumors while potentially acting upstream of Blimp-1, linking it to both tissue residency and anti-tumor immunity (28). Beyond these core factors, tissue-specific transcription factors further refine TRM identity; Hic1 governs intestinal TRM differentiation, NOTCH-1 regulates metabolic adaptation in human TRM cells, and Id3 defines intestinal intraepithelial lymphocyte subsets with enhanced cytokine production and proliferative capacity. Collectively, these findings highlight a complex, context-dependent transcriptional landscape in which overlapping yet distinct regulatory programs are tailored to the unique demands of each tissue microenvironment (**Table 1**).

**Table 1:** TRM cells – functions, tissue specificity and key transcription factors.

Marker name	Expression pattern	Primary function
CD69	Constitutive on most TRM cells	Tissue retention by antagonizing S1PR1
CD103	Enriched in mucosal and barrier tissues on CD8+ TRM	Epithelial adhesion by binding to E-cadherin
Tissue	Marker(s)	Significance
Mucosal (Lung, Gut, Skin)	CD103, CD49a	Retention and adhesion to epithelial cells and extracellular matrix
Brain	PD-1, CXCR6	Chronic expression of PD-1 for immune regulation, CXCR6 for homing/retention
Liver	CXCR6	Homing and retention
Transcription Factor	Primary Role in CD8 TRM Cells	
Blimp-1	Tissue residency, granzyme B regulation, lung TRM formation	
Hobit	Tissue residency, maintenance, granzyme B regulation	
Runx3	Programming tissue residency	
Hic1	Intestine-specific TRM differentiation and maintenance	
NOTCH-1	Regulation of TRM metabolism	

## The peripheral nervous system: structure and neuroimmune crosstalk

Research on brain-resident memory T cells has revealed their “sensing and alarm” function within the central nervous system (CNS), where they interact with various neural and glial cells (29, 30). This suggests that TRM cells, as sentinels in their respective tissues, might also interact with local tissue components, including nerve cells, in the peripheral nervous system. The capacity for such interactions in the CNS implies a potential for analogous relationships in the PNS, where TRM cells could monitor their environment and respond to signals from or influence the function of peripheral nerves. PNS forms an extensive network of sensory, motor, and autonomic nerves innervating virtually all peripheral tissues (31, 32), placing it in direct anatomical proximity to resident immune cells including TRM cells. Structurally, peripheral nerves comprise axon bundles ensheathed by epineurium, perineurium, and endoneurium, supported by Schwann cells, the glia which maintain myelination and facilitate nerve regeneration, and satellite glial cells, increasingly recognized for their roles in neuroimmune signaling. This anatomical overlap forms the structural basis for bidirectional communication between the PNS and the immune system, a relationship central to the emerging field of peripheral neuroimmunology (33).

## Role of TRM cells in homeostasis and pathologies

The literature has identified context-specific phenotypic characteristics of TRM cells across a range of pathological settings (19, 34). In colorectal cancer, co-expression of CD8, CD103, and CD39 on activated TRM cells has been shown to uniquely predict survival in “immune-hot” tumors, while CD103^+^/CD8^+^ tumor-infiltrating lymphocytes more broadly represent a favorable prognostic marker in this malignancy (35, 36). Similarly, in breast cancer, CD103^+^ resident memory CD8^+^ T cells localized within cancer islands have been found to mediate improved survival outcomes. In glioblastoma, infiltrating CD8^+^ T cells are predominantly clonally expanded populations expressing granzyme K (GZMK), suggesting a distinct effector TRM subset in this aggressive brain tumor. Additionally, pancreatic islets from donors with type 2 diabetes have been shown to harbor CD8^+^ T cells with high expression of canonical residency markers CD69 and CD103, implicating TRM cells in the pathogenesis of metabolic disease.

Single-cell RNA sequencing and spatial transcriptomics have further refined the understanding of TRM biology across tissue contexts. In small intestinal intraepithelial lymphocytes, Id3 expression has been shown to define unique TRM populations with enhanced cytokine production and proliferative capacity (37). In oral lichen planus, erosive lesions demonstrate an expansion of TRM cells producing elevated pro-inflammatory cytokines, while in colorectal cancer, TRM-derived gene risk score signatures have been shown to correlate with prognosis and therapeutic response (38, 39). In the context of HIV infection, CD8^+^ CD103+CD69+ TRM cells are much reduced in infected individuals compared to the uninfected, although with hallmarks of exhaustion including elevated PD-1 expression and diminished IFN-γ production in the gingival mucosa (16). This study underscores the vulnerability of tissue-resident immunity to chronic viral infection (40–42).

In autoimmune neuropathies such as Guillain-Barré Syndrome (GBS), although the role of localized TRM-mediated autoimmune responses is unclear, CD8^+^ T cells are recognized contributors to disease pathogenesis, potentially driving nerve damage (43). TRM cells in the brain drive compartmentalized neuroinflammation in multiple sclerosis, with tissue-resident T cells in white matter lesions promoting inflammatory activity (34, 44). In diabetic peripheral neuropathy (DPN), CD8^+^ T cell cytotoxicity toward Schwann cells further suggests that tissue-resident populations may exacerbate demyelination and impaired nerve conduction (45–47).

Several mechanisms may underlie TRM-mediated regulation of peripheral nerve function. Neuroimmune crosstalk is mediated by neurotransmitters, neuropeptides such as CGRP, substance P, and VIP, as well as immune-derived cytokines and chemokines that reciprocally influence nerve and immune cell function (48). Critically, commensal-specific CD8^+^ cells in the skin localize near cutaneous nerve fibers and upregulate RAMP1, the CGRP receptor, suggesting direct responsiveness to neural signals and potentially contributing to neuroinflammation (49). Additionally, CD8^+^ T cells have demonstrated cytotoxicity toward Schwann cells in diabetic peripheral neuropathy and accumulate within peripheral nerves in neuropathy models, implicating tissue-resident CD8^+^ populations in nerve pathology (45, 47). Pro-inflammatory cytokines including IFN-γ and TNF-α can induce neuroinflammation, demyelination, and nerve damage, while chemokines such as CXCL9 and CXCL10 recruit additional immune cells to the nerve microenvironment. Direct cytotoxic interactions via granzyme B and perforin could disrupt Schwann cell integrity and impair impulse conduction, while neuropeptide receptor expression on TRM cells enables neural modulation of local immune activity. Finally, TRM-derived mediators may indirectly regulate neurotrophic factors such as NGF and BDNF, influencing peripheral nerve maintenance, repair, and survival (50). Evidence in the literature also implicates dysregulated TRM cell activity in a range of peripheral nerve pathologies (**Table 2**).

**Table 2:** Potential mechanisms of TRM regulation of peripheral nerves.

Mechanism	Key signaling molecules involved	Potential effects on peripheral nerves
Cytokine Signaling	IFN-γ, TNF-α, Chemokines	Inflammation, nerve damage, demyelination, recruitment of other immune cells
Direct Cell Contact	Granzymes, Perforin, Surface Receptors/Ligands	Cytotoxicity towards Schwann cells, modulation of nerve cell activity or survival
Neuropeptide Interactions	CGRP, VIP, Substance P	Modulation of TRM cell activity by nerves, potential influence on neuropeptide signaling in nerves
Growth Factor Modulation	NGF, BDNF, Cytokines	Indirect regulation of nerve maintenance, repair, and survival

## TRM dysregulation during mucosal immune dysfunction in potential PNS inflammation

Parallels drawn from CNS-resident memory T cell involvement in neuroinflammatory and neurodegenerative diseases (34), suggest analogous pathological roles may exist in the PNS. Notably, in the context of viral infections affecting the PNS and mucosa, TRM cells, while crucial for viral clearance, their inflammatory phenotype, dysregulation or attrition, could potentially cause collateral nerve damage as part of the immune response (51, 52).

In the context of chronic HIV infection, mucosal immune dysregulation is compounded by oral microbial dysbiosis, with elevated *Fusobacteria* abundance shown to positively correlate with salivary polyamine levels in people living with HIV (PLWH) (16) (**Figure 1A**). Polyamines play a critical mechanistic role in suppressing TRM cell function through EIF5A hypusination, which reduces expression of IFN-γ and key TRM signature markers (16). Studies suggest that IFN-γ itself provides a feed-forward mechanism promoting TRM cell induction and proliferation, making its polyamine-dependent repression particularly detrimental to mucosal tissue-resident immunity (53). These results imply that dysbiotic microbiota, especially *Fusobacterium* as a substantial source of immunomodulatory polyamines in PLWH (54, 55). The additive effects of *Fusobacterium nucleatum* (FN) and HIV significantly diminish TRM-like cells, mirroring the reduced TRM cell frequency observed in the oral mucosa of PLWH (16). In addition, polyamines contribute to regulatory T cell (Treg) dysregulation (16), suggesting that chronic infection and dysbiosis drive coordinated immuno-metabolic dysfunction and residual mucosal inflammation, in the mucosal surface in HIV-infected population through modulating multiple immune cells (16, 55–58). Since Treg cells play a pivotal role in mitigating neuropathic pain by suppressing Th1-mediated immune responses at sites of nerve injury (46), TRM cell attrition along with Treg dysfunction and antigen-independent immune hyperactivation during chronic HIV infection could be a potential contributor to PNS inflammation, although direct experimental evidence remains limited. We further propose that PNS neuroinflammation may arise through one or more of three inflammatory pathways involving TRM cells: (1) cytokine-mediated signaling, (2) direct cytotoxicity, and (3) neurotransmitter-mediated signaling (**Figure 1B**). Furthermore, the upregulation of RAMP1- the receptor for the neuropeptide CGRP, on CD8^+^ T cells localized near cutaneous nerve fibers provides direct evidence of a communication pathway between TRM cells and peripheral nerves in the skin, highlighting the capacity for bidirectional neuroimmune regulation. Additional indirect interactions may also involve nerve growth factors that might be modulated by TRM cells, but the mechanistic details are limited (**Table 2**). Thus, the literature identifies several critical directions for advancing understanding of TRM-nerve interactions. Elucidating the precise molecular mechanisms governing communication between TRM cells and both neurons and glial cells is recognized as essential, alongside determining the functional consequences for nerve conduction velocity, pain perception, and autonomic regulation. Furthermore, the specific TRM cell subsets that are preferentially affected during chronic HIV infection have yet to be clearly defined.

**Figure 1:**
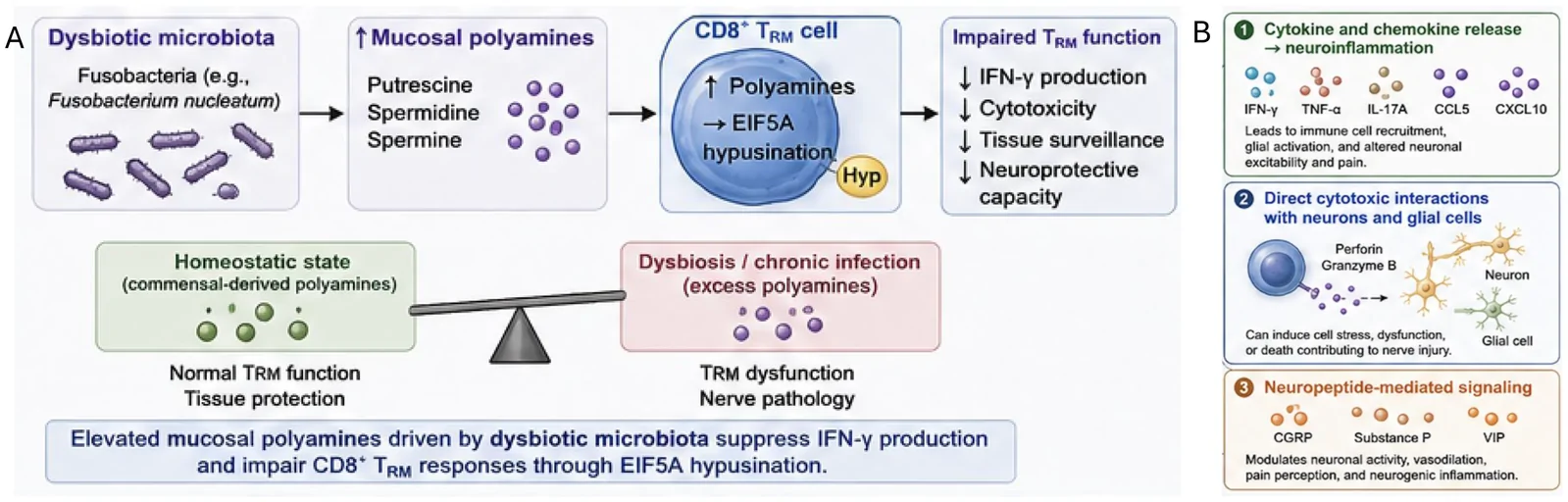
Mucosal dysfunction in triggering PNS neuroinflammation. **(A)** In the context of chronic HIV infection, mucosal immune dysfunction is compounded by oral microbial dysbiosis, with increased *Fusobacteria* abundance positively correlating with salivary putrescine levels in people living with HIV (PLWH). The combined effects of *Fusobacterium nucleatum* (FN) and HIV significantly reduce TRM. TRM cell attrition, together with Treg dysfunction and antigen-independent immune hyperactivation during chronic HIV infection, may contribute to PNS inflammation, although direct experimental evidence remains limited. **(B)** PNS neuroinflammation may arise through one or more of three direct pathways in TRM cells in the context of mucosal infection and inflammation: (1) cytokine-mediated signaling, (2) direct cytotoxicity, and (3) neurotransmitter-mediated signaling. (Artificial intelligence was used to draw some parts of the images in this figure).

Also, these studies highlight the broader pathological implications of TRM dysregulation of tumor immunity in PLWH (6, 16). The diminished frequency of CXCR6^+^CD8^+^ TRM cells observed in HIV infection may impair local tumor immunosurveillance. Given the established roles of both polyamines and *Fusobacterium nucleatum* in cancer pathogenesis, *Fusobacteria*-driven TRM decline may contribute to the increased cancer predisposition documented in PLWH (16, 59–65). Future investigations employing single-cell RNA sequencing are identified as essential for precisely defining TRM subset phenotypes within the mucosa. Also further systematic studies are required to clarify the impact of antiretroviral therapy on microbiome composition, the HIV-reactivity of TRM cells, and the consequences of chronic HIV infection for peripheral nervous system in the context of changes in mucosal tissues.

## Discussion

The available evidence suggests that TRM cells may regulate peripheral nerve function through multiple mechanisms, including cytokine and chemokine secretion that modulates neuroinflammation, direct cytotoxic interactions with neurons and glial cells, and neuropeptide-mediated signaling. Accordingly, dysregulation of TRM cells, particularly during chronic infection and microbial dysbiosis, may contribute to peripheral nerve pathology while also promoting broader immunometabolic dysfunction. In chronic HIV infection, this dysregulation may be amplified by oral microbial dysbiosis, including increased *Fusobacteria* abundance and its positive association with salivary putrescine levels in PLWH. Elevated mucosal polyamines can suppress IFN-γ production and impair TRM responses through EIF5A hypusination, thereby disrupting the homeostatic balance normally supported by commensal-derived polyamine metabolism. These may collectively contribute to PNS inflammation, although direct experimental evidence remains limited (**Figure 1**).

Conceptually, mucosal inflammation and associated PNS neuroinflammation may be linked through three principal TRM-mediated pathways (**Figure 1**): (1) cytokine- and chemokine-mediated signaling, (2) direct cytotoxicity toward neural cells, and (3) neurotransmitter- or neuropeptide-mediated signaling. These pathways provide a potential mechanistic framework connecting tissue-resident immunity, mucosal dysbiosis, and peripheral nerve dysfunction.

Future studies are needed to define the specific TRM subsets and molecular programs that contribute to the initiation and progression of peripheral neuropathy and to determine whether therapeutic modulation of TRM cells or the polyamine pathway can restore immune homeostasis and improve peripheral nerve function in different mucosal environments. In particular, identifying TRM markers and gene signatures associated with tissue residency and distinct functional states may provide new diagnostic and therapeutic opportunities. More broadly, defining the bidirectional interactions between tissue-resident immunity and the peripheral nervous system could reveal new strategies for treating neurological and chronic inflammatory diseases, including HIV-associated complications.
